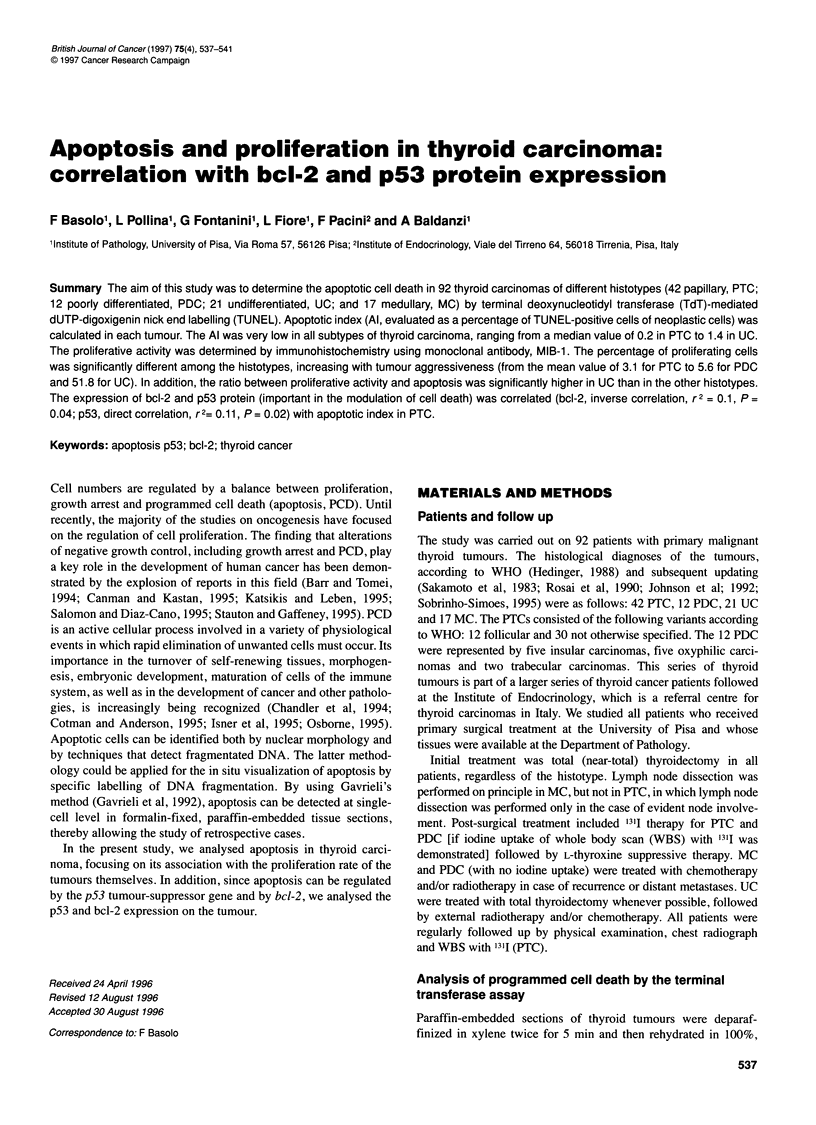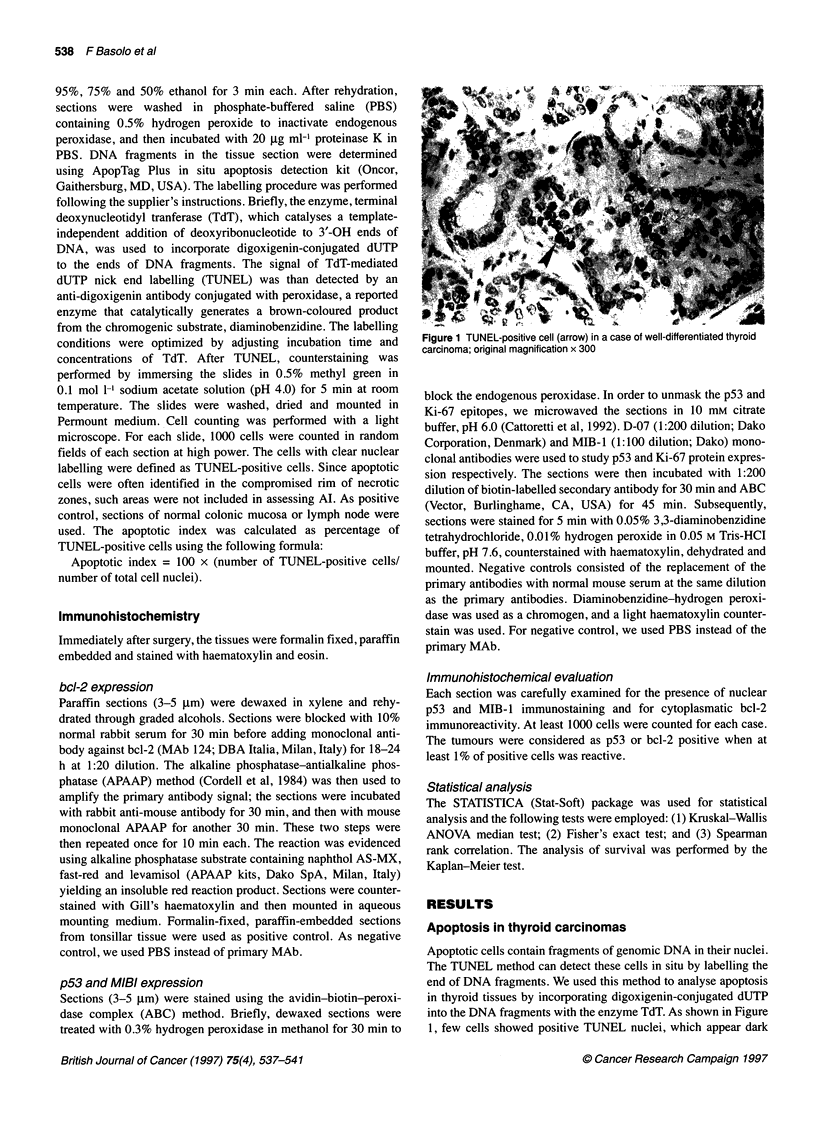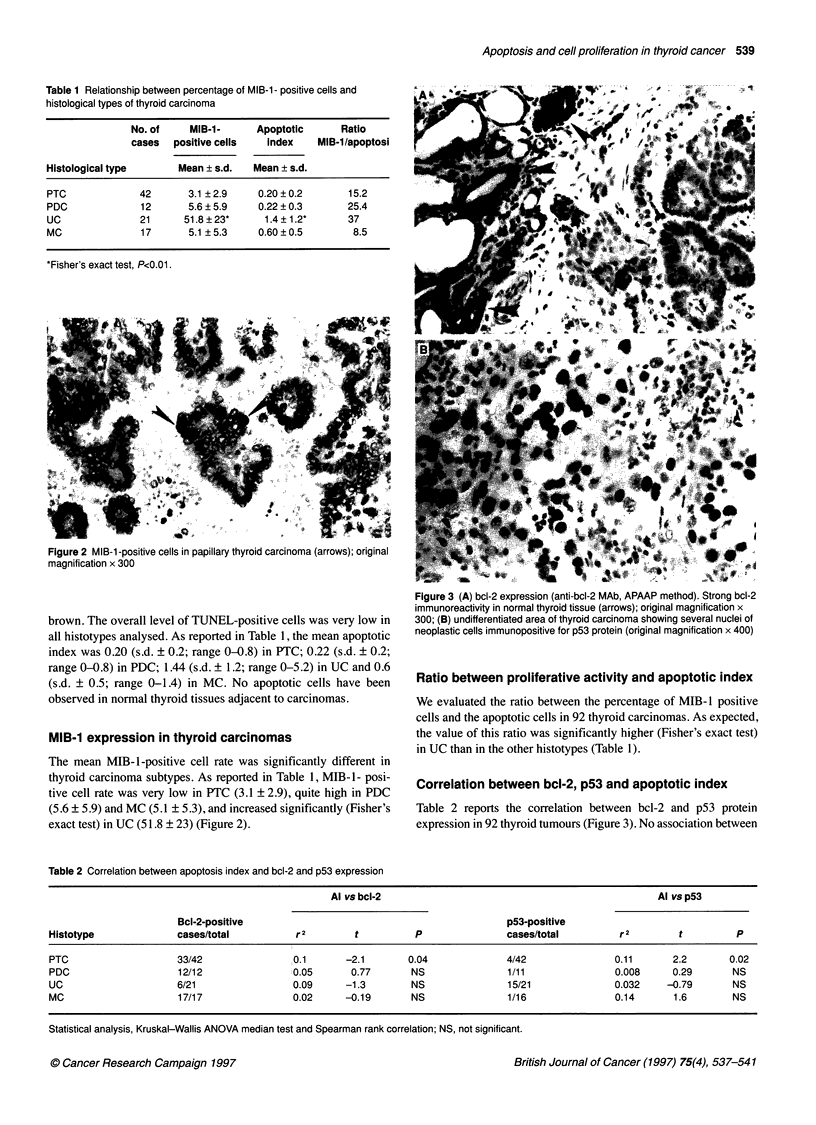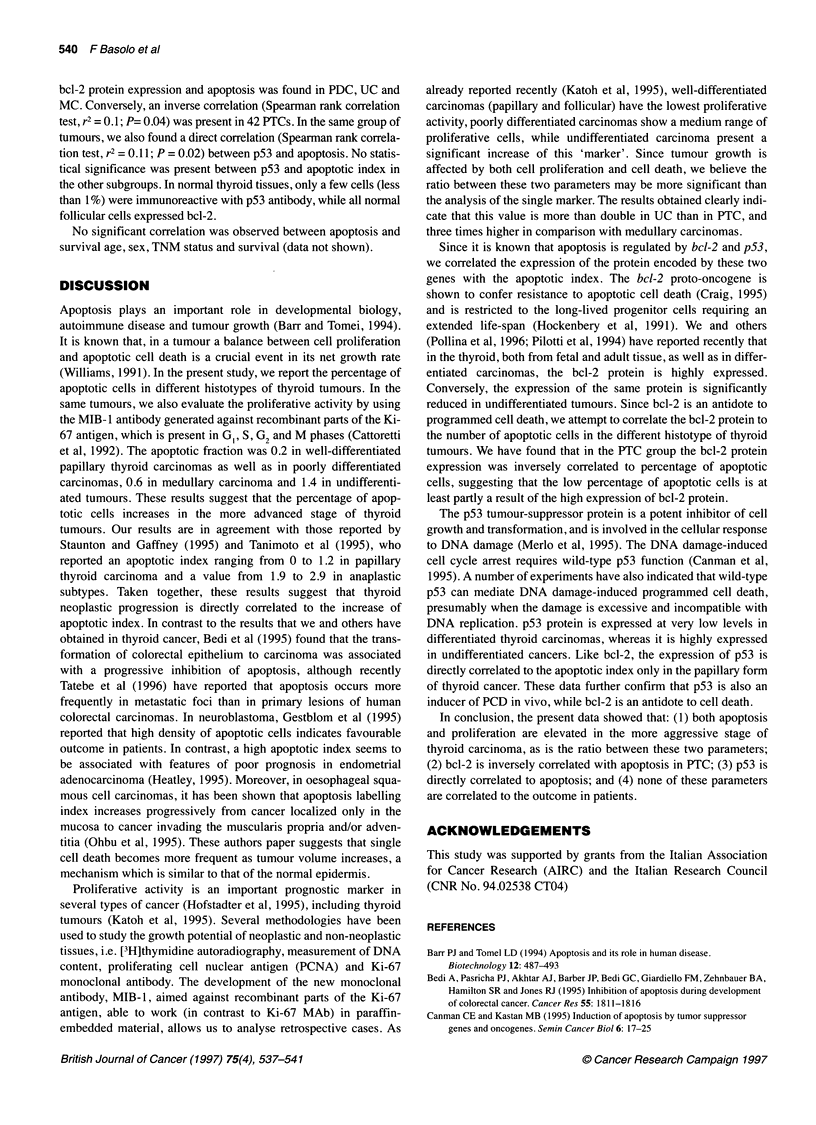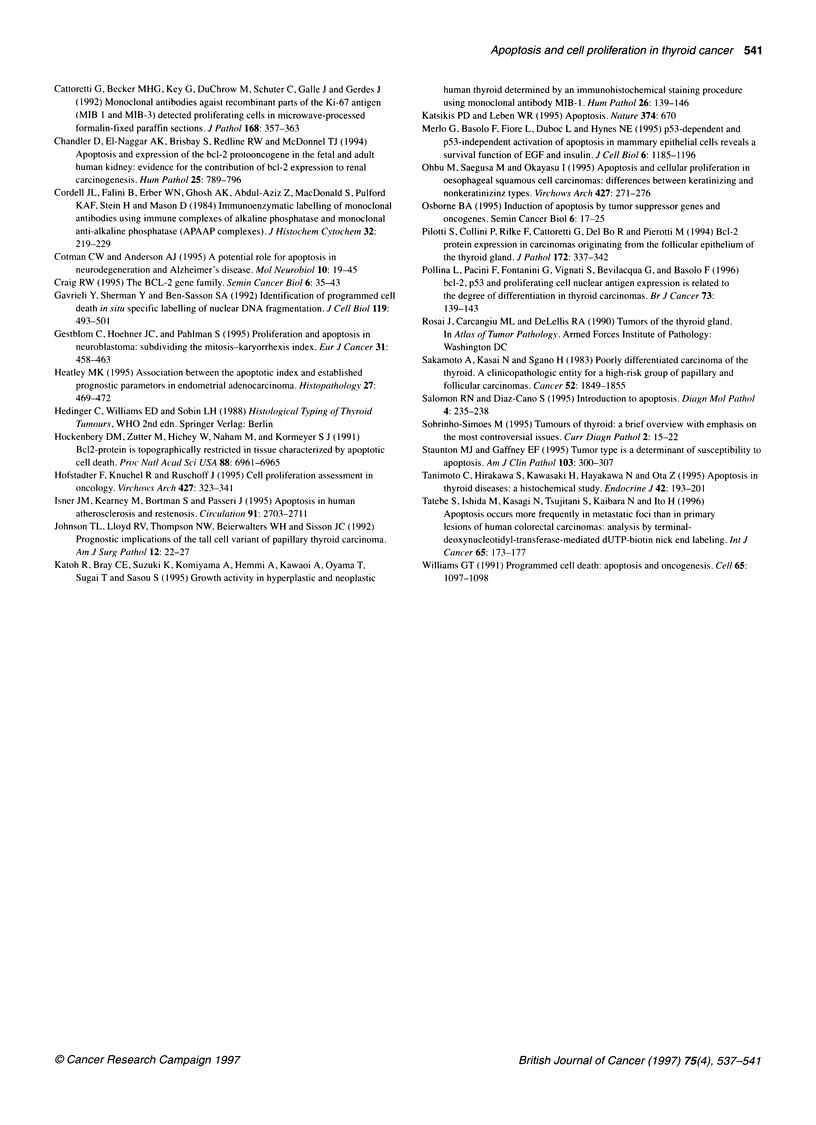# Apoptosis and proliferation in thyroid carcinoma: correlation with bcl-2 and p53 protein expression.

**DOI:** 10.1038/bjc.1997.93

**Published:** 1997

**Authors:** F. Basolo, L. Pollina, G. Fontanini, L. Fiore, F. Pacini, A. Baldanzi

**Affiliations:** Institute of Pathology, University of Pisa, Italy.

## Abstract

**Images:**


British Journal of Cancer (1997) 75(4), 537–541
© 1997 Cancer Research Campaign

### Apoptosis and proliferation in thyroid carcinoma: correlation with bcl-2 and p53 protein expression

F Basolo[1], L Pollina[1], G Fontanini[1], L Fiore[1], F Pacini[2] and A Baldanzi[1]

[1]Institute of Pathology, University of Pisa, Via Roma 57, 56126 Pisa; [2]Institute of Endocrinology, Viale del Tirreno 64, 56018 Tirrenia, Pisa, Italy

**Summary** The aim of this study was to determine the apoptotic cell death in 92 thyroid carcinomas of different histotypes (42 papillary, PTC; 12 poorly differentiated, PDC; 21 undifferentiated, UC; and 17 medullary, MC) by terminal deoxynucleotidyl transferase (TdT)-mediated dUTP-digoxigenin nick end labelling (TUNEL). Apoptotic index (AI, evaluated as a percentage of TUNEL-positive cells of neoplastic cells) was calculated in each tumour. The AI was very low in all subtypes of thyroid carcinoma, ranging from a median value of 0.2 in PTC to 1.4 in UC. The proliferative activity was determined by immunohistochemistry using monoclonal antibody, MIB-1. The percentage of proliferating cells was significantly different among the histotypes, increasing with tumour aggressiveness (from the mean value of 3.1 for PTC to 5.6 for PDC and 51.8 for UC). In addition, the ratio between proliferative activity and apoptosis was significantly higher in UC than in the other histotypes. The expression of bcl-2 and p53 protein (important in the modulation of cell death) was correlated (bcl-2, inverse correlation, $r^2 = 0.1$, $P = 0.04$; p53, direct correlation, $r^2 = 0.11$, $P = 0.02$) with apoptotic index in PTC.

**Keywords:** apoptosis p53; bcl-2; thyroid cancer

Cell numbers are regulated by a balance between proliferation, growth arrest and programmed cell death (apoptosis, PCD). Until recently, the majority of the studies on oncogenesis have focused on the regulation of cell proliferation. The finding that alterations of negative growth control, including growth arrest and PCD, play a key role in the development of human cancer has been demonstrated by the explosion of reports in this field (Barr and Tomei, 1994; Canman and Kastan, 1995; Katsikis and Leben, 1995; Salomon and Diaz-Cano, 1995; Stauton and Gaffeney, 1995). PCD is an active cellular process involved in a variety of physiological events in which rapid elimination of unwanted cells must occur. Its importance in the turnover of self-renewing tissues, morphogenesis, embryonic development, maturation of cells of the immune system, as well as in the development of cancer and other pathologies, is increasingly being recognized (Chandler et al, 1994; Cotman and Anderson, 1995; Isner et al, 1995; Osborne, 1995). Apoptotic cells can be identified both by nuclear morphology and by techniques that detect fragmentated DNA. The latter methodology could be applied for the in situ visualization of apoptosis by specific labelling of DNA fragmentation. By using Gavrieli's method (Gavrieli et al, 1992), apoptosis can be detected at single-cell level in formalin-fixed, paraffin-embedded tissue sections, thereby allowing the study of retrospective cases.

In the present study, we analysed apoptosis in thyroid carcinoma, focusing on its association with the proliferation rate of the tumours themselves. In addition, since apoptosis can be regulated by the *p53* tumour-suppressor gene and by *bcl-2*, we analysed the p53 and bcl-2 expression on the tumour.

*Received 24 April 1996*
*Revised 12 August 1996*
*Accepted 30 August 1996*

*Correspondence to:* F Basolo

### MATERIALS AND METHODS

### Patients and follow up

The study was carried out on 92 patients with primary malignant thyroid tumours. The histological diagnoses of the tumours, according to WHO (Hedinger, 1988) and subsequent updating (Sakamoto et al, 1983; Rosai et al, 1990; Johnson et al; 1992; Sobrinho-Simoes, 1995) were as follows: 42 PTC, 12 PDC, 21 UC and 17 MC. The PTCs consisted of the following variants according to WHO: 12 follicular and 30 not otherwise specified. The 12 PDC were represented by five insular carcinomas, five oxyphilic carcinomas and two trabecular carcinomas. This series of thyroid tumours is part of a larger series of thyroid cancer patients followed at the Institute of Endocrinology, which is a referral centre for thyroid carcinomas in Italy. We studied all patients who received primary surgical treatment at the University of Pisa and whose tissues were available at the Department of Pathology.

Initial treatment was total (near-total) thyroidectomy in all patients, regardless of the histotype. Lymph node dissection was performed on principle in MC, but not in PTC, in which lymph node dissection was performed only in the case of evident node involvement. Post-surgical treatment included $^{131}I$ therapy for PTC and PDC [if iodine uptake of whole body scan (WBS) with $^{131}I$ was demonstrated] followed by L-thyroxine suppressive therapy. MC and PDC (with no iodine uptake) were treated with chemotherapy and/or radiotherapy in case of recurrence or distant metastases. UC were treated with total thyroidectomy whenever possible, followed by external radiotherapy and/or chemotherapy. All patients were regularly followed up by physical examination, chest radiograph and WBS with $^{131}I$ (PTC).

### Analysis of programmed cell death by the terminal transferase assay

Paraffin-embedded sections of thyroid tumours were deparaffinized in xylene twice for 5 min and then rehydrated in 100%,

95%, 75% and 50% ethanol for 3 min each. After rehydration, sections were washed in phosphate-buffered saline (PBS) containing 0.5% hydrogen peroxide to inactivate endogenous peroxidase, and then incubated with 20 μg $ml^{-1}$ proteinase K in PBS. DNA fragments in the tissue section were determined using ApopTag Plus in situ apoptosis detection kit (Oncor, Gaithersburg, MD, USA). The labelling procedure was performed following the supplier's instructions. Briefly, the enzyme, terminal deoxynucleotidyl tranferase (TdT), which catalyses a template-independent addition of deoxyribonucleotide to 3′-OH ends of DNA, was used to incorporate digoxigenin-conjugated dUTP to the ends of DNA fragments. The signal of TdT-mediated dUTP nick end labelling (TUNEL) was than detected by an anti-digoxigenin antibody conjugated with peroxidase, a reported enzyme that catalytically generates a brown-coloured product from the chromogenic substrate, diaminobenzidine. The labelling conditions were optimized by adjusting incubation time and concentrations of TdT. After TUNEL, counterstaining was performed by immersing the slides in 0.5% methyl green in 0.1 mol $l^{-1}$ sodium acetate solution (pH 4.0) for 5 min at room temperature. The slides were washed, dried and mounted in Permount medium. Cell counting was performed with a light microscope. For each slide, 1000 cells were counted in random fields of each section at high power. The cells with clear nuclear labelling were defined as TUNEL-positive cells. Since apoptotic cells were often identified in the compromised rim of necrotic zones, such areas were not included in assessing AI. As positive control, sections of normal colonic mucosa or lymph node were used. The apoptotic index was calculated as percentage of TUNEL-positive cells using the following formula:

Apoptotic index = 100 × (number of TUNEL-positive cells/ number of total cell nuclei).

### Immunohistochemistry

Immediately after surgery, the tissues were formalin fixed, paraffin embedded and stained with haematoxylin and eosin.

### *bcl-2 expression*

Paraffin sections (3–5 μm) were dewaxed in xylene and rehydrated through graded alcohols. Sections were blocked with 10% normal rabbit serum for 30 min before adding monoclonal antibody against bcl-2 (MAb 124; DBA Italia, Milan, Italy) for 18–24 h at 1:20 dilution. The alkaline phosphatase–antialkaline phosphatase (APAAP) method (Cordell et al, 1984) was then used to amplify the primary antibody signal; the sections were incubated with rabbit anti-mouse antibody for 30 min, and then with mouse monoclonal APAAP for another 30 min. These two steps were then repeated once for 10 min each. The reaction was evidenced using alkaline phosphatase substrate containing naphthol AS-MX, fast-red and levamisol (APAAP kits, Dako SpA, Milan, Italy) yielding an insoluble red reaction product. Sections were counterstained with Gill's haematoxylin and then mounted in aqueous mounting medium. Formalin-fixed, paraffin-embedded sections from tonsillar tissue were used as positive control. As negative control, we used PBS instead of primary MAb.

### *p53 and MIBI expression*

Sections (3–5 μm) were stained using the avidin–biotin–peroxidase complex (ABC) method. Briefly, dewaxed sections were treated with 0.3% hydrogen peroxidase in methanol for 30 min to block the endogenous peroxidase. In order to unmask the p53 and Ki-67 epitopes, we microwaved the sections in 10 mM citrate buffer, pH 6.0 (Cattoretti et al, 1992). D-07 (1:200 dilution; Dako Corporation, Denmark) and MIB-1 (1:100 dilution; Dako) monoclonal antibodies were used to study p53 and Ki-67 protein expression respectively. The sections were then incubated with 1:200 dilution of biotin-labelled secondary antibody for 30 min and ABC (Vector, Burlinghame, CA, USA) for 45 min. Subsequently, sections were stained for 5 min with 0.05% 3,3-diaminobenzidine tetrahydrochloride, 0.01% hydrogen peroxide in 0.05 M Tris-HCI buffer, pH 7.6, counterstained with haematoxylin, dehydrated and mounted. Negative controls consisted of the replacement of the primary antibodies with normal mouse serum at the same dilution as the primary antibodies. Diaminobenzidine–hydrogen peroxidase was used as a chromogen, and a light haematoxylin counterstain was used. For negative control, we used PBS instead of the primary MAb.

**Figure 1** TUNEL-positive cell (arrow) in a case of well-differentiated thyroid carcinoma; original magnification × 300

### *Immunohistochemical evaluation*

Each section was carefully examined for the presence of nuclear p53 and MIB-1 immunostaining and for cytoplasmatic bcl-2 immunoreactivity. At least 1000 cells were counted for each case. The tumours were considered as p53 or bcl-2 positive when at least 1% of positive cells was reactive.

### *Statistical analysis*

The STATISTICA (Stat-Soft) package was used for statistical analysis and the following tests were employed: (1) Kruskal–Wallis ANOVA median test; (2) Fisher's exact test; and (3) Spearman rank correlation. The analysis of survival was performed by the Kaplan–Meier test.

### RESULTS

### Apoptosis in thyroid carcinomas

Apoptotic cells contain fragments of genomic DNA in their nuclei. The TUNEL method can detect these cells in situ by labelling the end of DNA fragments. We used this method to analyse apoptosis in thyroid tissues by incorporating digoxigenin-conjugated dUTP into the DNA fragments with the enzyme TdT. As shown in Figure 1, few cells showed positive TUNEL nuclei, which appear dark

© Cancer Research Campaign 1997

**Table 1** Relationship between percentage of MIB-1- positive cells and histological types of thyroid carcinoma

| Histological type | No. of cases | MIB-1-positive cells Mean ± s.d. | Apoptotic index Mean ± s.d. | Ratio MIB-1/apoptosi |
|---|---|---|---|---|
| PTC | 42 | 3.1 ± 2.9 | 0.20 ± 0.2 | 15.2 |
| PDC | 12 | 5.6 ± 5.9 | 0.22 ± 0.3 | 25.4 |
| UC | 21 | 51.8 ± 23* | 1.4 ± 1.2* | 37 |
| MC | 17 | 5.1 ± 5.3 | 0.60 ± 0.5 | 8.5 |

*Fisher's exact test, $P$<0.01.

**Figure 2** MIB-1-positive cells in papillary thyroid carcinoma (arrows); original magnification × 300

brown. The overall level of TUNEL-positive cells was very low in all histotypes analysed. As reported in Table 1, the mean apoptotic index was 0.20 (s.d. ± 0.2; range 0–0.8) in PTC; 0.22 (s.d. ± 0.2; range 0–0.8) in PDC; 1.44 (s.d. ± 1.2; range 0–5.2) in UC and 0.6 (s.d. ± 0.5; range 0–1.4) in MC. No apoptotic cells have been observed in normal thyroid tissues adjacent to carcinomas.

### MIB-1 expression in thyroid carcinomas

The mean MIB-1-positive cell rate was significantly different in thyroid carcinoma subtypes. As reported in Table 1, MIB-1- positive cell rate was very low in PTC (3.1 ± 2.9), quite high in PDC (5.6 ± 5.9) and MC (5.1 ± 5.3), and increased significantly (Fisher's exact test) in UC (51.8 ± 23) (Figure 2).

**Figure 3** (**A**) bcl-2 expression (anti-bcl-2 MAb, APAAP method). Strong bcl-2 immunoreactivity in normal thyroid tissue (arrows); original magnification × 300; (**B**) undifferentiated area of thyroid carcinoma showing several nuclei of neoplastic cells immunopositive for p53 protein (original magnification × 400)

### Ratio between proliferative activity and apoptotic index

We evaluated the ratio between the percentage of MIB-1 positive cells and the apoptotic cells in 92 thyroid carcinomas. As expected, the value of this ratio was significantly higher (Fisher's exact test) in UC than in the other histotypes (Table 1).

### Correlation between bcl-2, p53 and apoptotic index

Table 2 reports the correlation between bcl-2 and p53 protein expression in 92 thyroid tumours (Figure 3). No association between

**Table 2** Correlation between apoptosis index and bcl-2 and p53 expression

| Histotype | Bcl-2-positive cases/total | AI *vs* bcl-2 $r^2$ | AI *vs* bcl-2 $t$ | AI *vs* bcl-2 $P$ | p53-positive cases/total | AI *vs* p53 $r^2$ | AI *vs* p53 $t$ | AI *vs* p53 $P$ |
|---|---|---|---|---|---|---|---|---|
| PTC | 33/42 | 0.1 | –2.1 | 0.04 | 4/42 | 0.11 | 2.2 | 0.02 |
| PDC | 12/12 | 0.05 | 0.77 | NS | 1/11 | 0.008 | 0.29 | NS |
| UC | 6/21 | 0.09 | –1.3 | NS | 15/21 | 0.032 | –0.79 | NS |
| MC | 17/17 | 0.02 | –0.19 | NS | 1/16 | 0.14 | 1.6 | NS |

Statistical analysis, Kruskal–Wallis ANOVA median test and Spearman rank correlation; NS, not significant.

© Cancer Research Campaign 1997

bcl-2 protein expression and apoptosis was found in PDC, UC and MC. Conversely, an inverse correlation (Spearman rank correlation test, $r^2 = 0.1$; $P= 0.04$) was present in 42 PTCs. In the same group of tumours, we also found a direct correlation (Spearman rank correlation test, $r^2 = 0.11$; $P = 0.02$) between p53 and apoptosis. No statistical significance was present between p53 and apoptotic index in the other subgroups. In normal thyroid tissues, only a few cells (less than 1%) were immunoreactive with p53 antibody, while all normal follicular cells expressed bcl-2.

No significant correlation was observed between apoptosis and survival age, sex, TNM status and survival (data not shown).

### DISCUSSION

Apoptosis plays an important role in developmental biology, autoimmune disease and tumour growth (Barr and Tomei, 1994). It is known that, in a tumour a balance between cell proliferation and apoptotic cell death is a crucial event in its net growth rate (Williams, 1991). In the present study, we report the percentage of apoptotic cells in different histotypes of thyroid tumours. In the same tumours, we also evaluate the proliferative activity by using the MIB-1 antibody generated against recombinant parts of the Ki-67 antigen, which is present in $G_1$, S, $G_2$ and M phases (Cattoretti et al, 1992). The apoptotic fraction was 0.2 in well-differentiated papillary thyroid carcinomas as well as in poorly differentiated carcinomas, 0.6 in medullary carcinoma and 1.4 in undifferentiated tumours. These results suggest that the percentage of apoptotic cells increases in the more advanced stage of thyroid tumours. Our results are in agreement with those reported by Staunton and Gaffney (1995) and Tanimoto et al (1995), who reported an apoptotic index ranging from 0 to 1.2 in papillary thyroid carcinoma and a value from 1.9 to 2.9 in anaplastic subtypes. Taken together, these results suggest that thyroid neoplastic progression is directly correlated to the increase of apoptotic index. In contrast to the results that we and others have obtained in thyroid cancer, Bedi et al (1995) found that the transformation of colorectal epithelium to carcinoma was associated with a progressive inhibition of apoptosis, although recently Tatebe et al (1996) have reported that apoptosis occurs more frequently in metastatic foci than in primary lesions of human colorectal carcinomas. In neuroblastoma, Gestblom et al (1995) reported that high density of apoptotic cells indicates favourable outcome in patients. In contrast, a high apoptotic index seems to be associated with features of poor prognosis in endometrial adenocarcinoma (Heatley, 1995). Moreover, in oesophageal squamous cell carcinomas, it has been shown that apoptosis labelling index increases progressively from cancer localized only in the mucosa to cancer invading the muscularis propria and/or adventitia (Ohbu et al, 1995). These authors paper suggests that single cell death becomes more frequent as tumour volume increases, a mechanism which is similar to that of the normal epidermis.

Proliferative activity is an important prognostic marker in several types of cancer (Hofstadter et al, 1995), including thyroid tumours (Katoh et al, 1995). Several methodologies have been used to study the growth potential of neoplastic and non-neoplastic tissues, i.e. [$^3$H]thymidine autoradiography, measurement of DNA content, proliferating cell nuclear antigen (PCNA) and Ki-67 monoclonal antibody. The development of the new monoclonal antibody, MIB-1, aimed against recombinant parts of the Ki-67 antigen, able to work (in contrast to Ki-67 MAb) in paraffin-embedded material, allows us to analyse retrospective cases. As already reported recently (Katoh et al, 1995), well-differentiated carcinomas (papillary and follicular) have the lowest proliferative activity, poorly differentiated carcinomas show a medium range of proliferative cells, while undifferentiated carcinoma present a significant increase of this 'marker'. Since tumour growth is affected by both cell proliferation and cell death, we believe the ratio between these two parameters may be more significant than the analysis of the single marker. The results obtained clearly indicate that this value is more than double in UC than in PTC, and three times higher in comparison with medullary carcinomas.

Since it is known that apoptosis is regulated by *bcl-2* and *p53*, we correlated the expression of the protein encoded by these two genes with the apoptotic index. The *bcl-2* proto-oncogene is shown to confer resistance to apoptotic cell death (Craig, 1995) and is restricted to the long-lived progenitor cells requiring an extended life-span (Hockenbery et al, 1991). We and others (Pollina et al, 1996; Pilotti et al, 1994) have reported recently that in the thyroid, both from fetal and adult tissue, as well as in differentiated carcinomas, the bcl-2 protein is highly expressed. Conversely, the expression of the same protein is significantly reduced in undifferentiated tumours. Since bcl-2 is an antidote to programmed cell death, we attempt to correlate the bcl-2 protein to the number of apoptotic cells in the different histotype of thyroid tumours. We have found that in the PTC group the bcl-2 protein expression was inversely correlated to percentage of apoptotic cells, suggesting that the low percentage of apoptotic cells is at least partly a result of the high expression of bcl-2 protein.

The p53 tumour-suppressor protein is a potent inhibitor of cell growth and transformation, and is involved in the cellular response to DNA damage (Merlo et al, 1995). The DNA damage-induced cell cycle arrest requires wild-type p53 function (Canman et al, 1995). A number of experiments have also indicated that wild-type p53 can mediate DNA damage-induced programmed cell death, presumably when the damage is excessive and incompatible with DNA replication. p53 protein is expressed at very low levels in differentiated thyroid carcinomas, whereas it is highly expressed in undifferentiated cancers. Like bcl-2, the expression of p53 is directly correlated to the apoptotic index only in the papillary form of thyroid cancer. These data further confirm that p53 is also an inducer of PCD in vivo, while bcl-2 is an antidote to cell death.

In conclusion, the present data showed that: (1) both apoptosis and proliferation are elevated in the more aggressive stage of thyroid carcinoma, as is the ratio between these two parameters; (2) bcl-2 is inversely correlated with apoptosis in PTC; (3) p53 is directly correlated to apoptosis; and (4) none of these parameters are correlated to the outcome in patients.

### ACKNOWLEDGEMENTS

This study was supported by grants from the Italian Association for Cancer Research (AIRC) and the Italian Research Council (CNR No. 94.02538 CT04)

### REFERENCES

Barr PJ and Tomel LD (1994) Apoptosis and its role in human disease. *Biotechnology* **12**: 487–493

Bedi A, Pasricha PJ, Akhtar AJ, Barber JP, Bedi GC, Giardiello FM, Zehnbauer BA, Hamilton SR and Jones RJ (1995) Inhibition of apoptosis during development of colorectal cancer. *Cancer Res* **55**: 1811–1816

Canman CE and Kastan MB (1995) Induction of apoptosis by tumor suppressor genes and oncogenes. *Semin Cancer Biol* **6**: 17–25

© Cancer Research Campaign 1997

Cattoretti G, Becker MHG, Key G, DuChrow M, Schuter C, Galle J and Gerdes J (1992) Monoclonal antibodies agaist recombinant parts of the Ki-67 antigen (MIB 1 and MIB-3) detected proliferating cells in microwave-processed formalin-fixed paraffin sections. *J Pathol* **168**: 357–363

Chandler D, El-Naggar AK, Brisbay S, Redline RW and McDonnel TJ (1994) Apoptosis and expression of the bcl-2 protooncogene in the fetal and adult human kidney: evidence for the contribution of bcl-2 expression to renal carcinogenesis. *Hum Pathol* **25**: 789–796

Cordell JL, Falini B, Erber WN, Ghosh AK, Abdul-Aziz Z, MacDonald S, Pulford KAF, Stein H and Mason D (1984) Immunoenzymatic labelling of monoclonal antibodies using immune complexes of alkaline phosphatase and monoclonal anti-alkaline phosphatase (APAAP complexes). *J Histochem Cytochem* **32**: 219–229

Cotman CW and Anderson AJ (1995) A potential role for apoptosis in neurodegeneration and Alzheimer's disease. *Mol Neurobiol* **10**: 19–45

Craig RW (1995) The BCL-2 gene family. *Semin Cancer Biol* **6**: 35–43

Gavrieli Y, Sherman Y and Ben-Sasson SA (1992) Identification of programmed cell death *in situ* specific labelling of nuclear DNA fragmentation. *J Cell Biol* **119**: 493–501

Gestblom C, Hoehner JC, and Pahlman S (1995) Proliferation and apoptosis in neuroblastoma: subdividing the mitosis–karyorrhexis index. *Eur J Cancer* **31**: 458–463

Heatley MK (1995) Association between the apoptotic index and established prognostic parametors in endometrial adenocarcinoma. *Histopathology* **27**: 469–472

Hedinger C, Williams ED and Sobin LH (1988) *Histological Typing of Thyroid Tumours*, WHO 2nd edn. Springer Verlag: Berlin

Hockenbery DM, Zutter M, Hichey W, Naham M, and Kormeyer S J (1991) Bcl2-protein is topographically restricted in tissue characterized by apoptotic cell death. *Proc Natl Acad Sci USA* **88**: 6961–6965

Hofstadter F, Knuchel R and Ruschoff J (1995) Cell proliferation assessment in oncology. *Virchows Arch* **427**: 323–341

Isner JM, Kearney M, Bortman S and Passeri J (1995) Apoptosis in human atherosclerosis and restenosis. *Circulation* **91**: 2703–2711

Johnson TL, Lloyd RV, Thompson NW, Beierwalters WH and Sisson JC (1992) Prognostic implications of the tall cell variant of papillary thyroid carcinoma. *Am J Surg Pathol* **12**: 22–27

Katoh R, Bray CE, Suzuki K, Komiyama A, Hemmi A, Kawaoi A, Oyama T, Sugai T and Sasou S (1995) Growth activity in hyperplastic and neoplastic human thyroid determined by an immunohistochemical staining procedure using monoclonal antibody MIB-1. *Hum Pathol* **26**: 139–146

Katsikis PD and Leben WR (1995) Apoptosis. *Nature* **374**: 670

Merlo G, Basolo F, Fiore L, Duboc L and Hynes NE (1995) p53-dependent and p53-independent activation of apoptosis in mammary epithelial cells reveals a survival function of EGF and insulin. *J Cell Biol* **6**: 1185–1196

Ohbu M, Saegusa M and Okayasu I (1995) Apoptosis and cellular proliferation in oesophageal squamous cell carcinomas: differences between keratinizing and nonkeratinizinz types. *Virchows Arch* **427**: 271–276

Osborne BA (1995) Induction of apoptosis by tumor suppressor genes and oncogenes. Semin Cancer Biol **6**: 17–25

Pilotti S, Collini P, Rilke F, Cattoretti G, Del Bo R and Pierotti M (1994) Bcl-2 protein expression in carcinomas originating from the follicular epithelium of the thyroid gland. *J Pathol* **172**: 337–342

Pollina L, Pacini F, Fontanini G, Vignati S, Bevilacqua G, and Basolo F (1996) bcl-2, p53 and proliferating cell nuclear antigen expression is related to the degree of differentiation in thyroid carcinomas. *Br J Cancer* **73**: 139–143

Rosai J, Carcangiu ML and DeLellis RA (1990) Tumors of the thyroid gland. In *Atlas of Tumor Pathology*. Armed Forces Institute of Pathology: Washington DC

Sakamoto A, Kasai N and Sgano H (1983) Poorly differentiated carcinoma of the thyroid. A clinicopathologic entity for a high-risk group of papillary and follicular carcinomas. *Cancer* **52**: 1849–1855

Salomon RN and Diaz-Cano S (1995) Introduction to apoptosis. *Diagn Mol Pathol* **4**: 235–238

Sobrinho-Simoes M (1995) Tumours of thyroid: a brief overview with emphasis on the most controversial issues. *Curr Diagn Pathol* **2**: 15–22

Staunton MJ and Gaffney EF (1995) Tumor type is a determinant of susceptibility to apoptosis. *Am J Clin Pathol* **103**: 300–307

Tanimoto C, Hirakawa S, Kawasaki H, Hayakawa N and Ota Z (1995) Apoptosis in thyroid diseases: a histochemical study. *Endocrine J* **42**: 193–201

Tatebe S, Ishida M, Kasagi N, Tsujitani S, Kaibara N and Ito H (1996) Apoptosis occurs more frequently in metastatic foci than in primary lesions of human colorectal carcinomas: analysis by terminal-deoxynucleotidyl-transferase-mediated dUTP-biotin nick end labeling. *Int J Cancer* **65**: 173–177

Williams GT (1991) Programmed cell death: apoptosis and oncogenesis. *Cell* **65**: 1097–1098

© Cancer Research Campaign 1997